# Blockchain for Modern Applications: A Survey

**DOI:** 10.3390/s22145274

**Published:** 2022-07-14

**Authors:** Moez Krichen, Meryem Ammi, Alaeddine Mihoub, Mutiq Almutiq

**Affiliations:** 1 Faculty of Computer Science and Information Technology, Albaha University, Alaqiq 65779, Saudi Arabia; mkreishan@bu.edu.sa or; 2 ReDCAD Laboratory, National School of Engineers of Sfax, University of Sfax, Sfax 3038, Tunisia; 3 Digital Forensics Department, Criminal Justice College, Naif Arab University for Security Sciences, Riyadh 14812, Saudi Arabia; mammi@nauss.edu.sa; 4 Department of Management Information Systems and Production Management, College of Business and Economics, Qassim University, P.O. Box 6640, Buraidah 51452, Saudi Arabia; a.mihoub@qu.edu.sa

**Keywords:** blockchain, review, finance, healthcare, information systems, wireless networks, Internet of Things (IoT), smart grids, governmental services, military/defense

## Abstract

Blockchain is a modern technology that has revolutionized the way society interacts and trades. It could be defined as a chain of blocks that stores information with digital signatures in a distributed and decentralized network. This technique was first adopted for the creation of digital cryptocurrencies, such as Bitcoin and Ethereum. However, research and industrial studies have recently focused on the opportunities that blockchain provides in various other application domains to take advantage of the main features of this technology, such as: decentralization, persistency, anonymity, and auditability. This paper reviews the use of blockchain in several interesting fields, namely: finance, healthcare, information systems, wireless networks, Internet of Things, smart grids, governmental services, and military/defense. In addition, our paper identifies the challenges to overcome, to guarantee better use of this technology.

## 1. Introduction

Blockchain is a revolutionary paradigm that has introduced new concepts into securely sharing data and information. This modern technology consists of a chain of blocks that allows to securely store all committed transactions using shared and distributed networks [1,2]. To fulfill this goal, several basic technologies are adopted, such as the cryptographic hash function, distributed consensus algorithms, and digital signatures. All transactions are carried out in a decentralized way, removing the need for any mediators to confirm and verify them [3]. Blockchain has some key characteristics [4], such as:

- Decentralization: In the blockchain, a transaction can be performed between any two entities/actors without the need for central authentication. As a result, the use of blockchain can dramatically cut server expenses while also alleviating performance constraints at the central server.
- Persistency: it is nearly impossible to tamper with the system because each transaction must be validated and recorded in blocks dispersed across the whole network.
- Anonymity: With a created address, each user can communicate with the blockchain network. Furthermore, a user could generate a large number of addresses in order to protect his/her identity. It is (worth mentioning that just a few blockchain implementations offer anonymity. The majority of them are pseudonymous).
- Auditability: users can easily check and trace prior records by accessing any node in the distributed network because each transaction is confirmed and stored with a timestamp.

Blockchain was initially proposed for supporting the well-known cryptocurrency, Bitcoin [5]. However, during the last few years, blockchain was adopted in several new fields far beyond cryptocurrencies [6], including healthcare [7], intelligent transportation [8], and Internet of Things (IoT) [9]. Indeed, thanks to its ability to increase fairness and transparency and to help organizations save money and time, this technology is influencing a wide range of industries [10], ranging from basic individual entertainment activities to the management of critical and sensitive affairs of governments and states.

**Figure 1:**
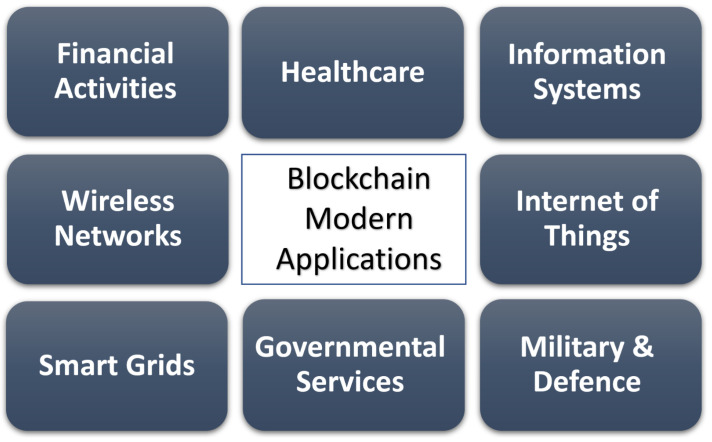
Blockchain application domains covered by this survey.

In this paper, we mainly focus on recent studies related to the incorporation of blockchain technology in modern applications, by comprehensively discussing the advantages and challenges related to the proposed solution. By doing so, we provide a survey on the use of blockchain in some modern applications (Figure 1 and Table 1):

- Financial Activities (Section 4);
- Healthcare (Section 5);
- Information systems (Section 6);
- Wireless networks (Section 7);
- Internet of Things (Section 8);
- Smart grids (Section 9);
- Governmental services (Section 10);
- Military and defense (Section 11).

For each domain aforementioned, we propose some related examples for the use of the blockchain technology while focusing on the corresponding benefits, limitations, and challenges. In Section 2, we present a quick summary of similar survey articles about blockchain technology that have been published between 2020 and 2022. In Section 3, an overview of the blockchain architecture is provided. Section 12 lists the main open challenges related to the use of blockchain technology. Section 13 presents a general conclusion of the paper.

## 2. Related Surveys

In this section, we provide a concise summary of nine related survey articles dealing with the use of blockchain technology in modern applications that have been published between 2020 and 2022.

An assessment of blockchain applications in smart grids with regard to cyber security perceptions and energy data protections was published in [33]. The authors discussed how big data and blockchain might help tackle major security challenges in smart grid scenarios. The researchers then identified a number of recent blockchain-based research papers that had been published in various journals, as well as examined security risks with smart grid technologies. They talked about a number of other recent practical designs, experiments, and products. Finally, they discussed some of the most pressing research issues as well as potential avenues for utilizing blockchain to address smart grid security challenges.

The writers of [34] conducted a survey and tutorial on blockchain applications in IoT systems. Based on the most important aspects, they suggested a blockchain taxonomy for IoT applications. They also looked at the most popular blockchain systems for IoT applications. They talked about how blockchain technology can be utilized to expand the range of IoT applications. Furthermore, they focused on new advancements and solutions for the IoT context. Finally, they discussed the obstacles and future research objectives for blockchain applications in IoT.

By assessing, arranging, and summarizing the literature, the authors of [35] offered a comprehensive overview of blockchain technology’s role in tackling supply chain and logistics-related concerns. The proposed study demonstrated that blockchain technology may transform the supply chain and logistics services into secure, flexible, trustworthy, and transparent operations. The advantages of blockchain technology in giving provenance and traceability to crucial products are highlighted through an imagined application scenario.

The research [36] provides an overview of blockchains, including their construction, consensus techniques, and other topics. It compares algorithms based on their usefulness and drawbacks. The importance of blockchains in the sectors of smart healthcare, smart grids, and smart financial systems is also discussed in this study. Overall, this paper provides an overview of the blockchain domain’s numerous protocols, algorithms, applications, difficulties, and potential.

The study provided in [37] focused on the potential applications of blockchain in future transportation systems that will be combined with connected and autonomous cars, in order to offer a general review of the current related literature and research activities on this subject. In addition, the writers focused on the problems, roadblocks, and future research areas associated with blockchain implementation in this context.

The writers of [38] provided an in-depth examination of blockchain technology’s evolution, architecture, development frameworks, and security concerns. They also gave a comparison of frameworks, a categorization of consensus methods, and an examination of security threats and cryptographic primitives employed in the blockchain. Finally, they discussed critical future possible extensions and open research issues that researchers may investigate in order to make more progress in this field. The authors took a broad approach in this research and did not focus on the use of blockchain in any specific domains.

The paper [39] provides a comprehensive overview of blockchain technology’s applications and use cases for securing and trusting smart systems. Readers of this article will gain a solid understanding of blockchain technology’s applications and use cases.

The authors of [40] proposed a complete overview of blockchain applications, architectures, methodologies, and research issues in Industry 4.0. They presented a blockchain reference architecture for smart manufacturing, which drove their discussions on how to deploy blockchain technology to smart factory and smart supply chain applications. The authors covered only a limited number of limitations, namely, throughput and scalability; system integration, and privacy and security.

The authors of [41] proposed a taxonomy that incorporates both technical and application information and could help academics construct blockchain-based multimedia copyright protection systems. The study also explored several technical issues and suggested future research directions.

A summary of the previous studied survey articles is provided in Table 2. By studying these survey articles, we noticed that most concentrated on a few application domains in each article and did not provide enough details about the issues and challenges faced in the considered fields. For this reason, our paper aimed to cover a larger number of application fields and provide more insights into the problems and difficulties encountered in these domains.

## 3. Blockchain Architecture

**Figure 2:**
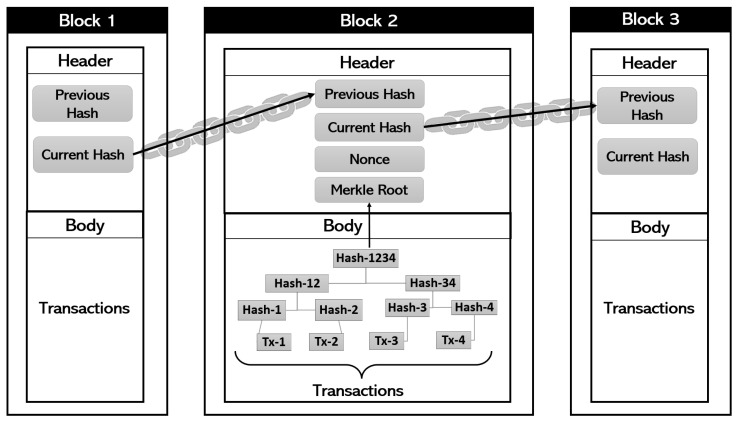
Blockchain general architecture.

A blockchain is a continuously expanding collection of data blocks linked together to form a long chain [42] as described in Figure 2. This network of connected data blocks represents a distributed ledger that is disseminated over a peer-to-peer network [43]. A distributed ledger contains a collection of digital data that are synced, replicated, distributed, and shared through a peer-to-peer network. Each device linked to the network maintains the latest version of the common ledger, i.e., each peer in the network has a copy of the ledger that is identical to the other. The ledger is mainly characterized by its safety, and the database can be expanded only by the addition of new blocks to the chain. Changes to records that have already been registered to the chain are computationally impossible. As a result, a primary benefit of the described distributed ledger is its decentralized nature. Indeed, there is no central authority that controls the ledger; however, each node updates its ledger when a new block is added to the blockchain, using a joint consensus mechanism [44]. Moreover, in the blockchain, and especially in the cryptocurrency networks, the authenticity of data is frequently verified by an asymmetric encryption technology known as public-key cryptography (PKC) [45]. In this technology, both the transmitter and receiver have a pair of keys consisting of a public key and a private one [46]. The private key is exclusively accessible to the nodes that created it, whereas the public key is spread rather freely throughout the network. The sender encrypts the data using the receiver’s public key. Since data are encrypted using the receiver’s public key, they can only be decrypted using the receiver’s private key. Furthermore, in the case of sending transactions on a blockchain network, a transaction is deemed complete only after it is digitally signed. Following that, the transaction is signed by the sender using his private key. For the receiver, the transaction’s authenticity i.e., the sender‘s’ identity, can be checked using the associated public key (belonging to the sender). This way, all transactions are automatically checked and authenticated by nodes and the network rejects any unauthenticated transactions. Please note that on a blockchain network, an authentic, mined transaction is irreversible [47].

Actually, it is difficult to alter the data contained in blocks thanks to the cryptographic qualities of the blockchain. Practically, the blocks are connected via a hash reference since each subsequent block carries the previous block’s hash value in addition to the actual block’s hash value (Figure 2). Generating a hash value is feasible through the use of a mathematical and sophisticated cryptographic hash algorithm, which accepts any input type and outputs a fixed-length number termed as the hash value. The primary characteristic of a hash function is that if a single fraction in the input is changed, the entire value in the output will be altered [48]. Consequently, if an attacker attempts to edit data in Block 1 (B1) for instance, the hash value of that block (B1) will be modified in the following block (B2), and so the intruder will have to modify the hash value of that block. Moreover, because B2 curries the hash of B1, any modification in the hash will alter the hash value of B2 in B3. As a result, if someone wants to modify a block, he or she must modify the data for all subsequent blocks on the blockchain. Additionally, even if the hash value of a block is known, calculating the hash function’s input is difficult due to the hash function’s non-invertible feature [46].

The next question is how to add new blocks to the network. Indeed, if we take the special case of the bitcoin cryptocurrency, there are particular types of nodes called “Miners” that are responsible for building new blocks in the chain [47]. The miner’s job is to update (from prior transactions) the records of the blockchain public ledger. Any network node could be a miner. It takes miners hours to create a new block because they must resolve a mathematical puzzle called “Proof of Work” (PoW). Several miners can work in parallel to add a new block. Nevertheless, only one miner can add a novel block at a moment. The first miner to solve the PoW problem can mine that new block. To address the mining PoW problem, huge computing power is needed. We could break down the whole process into multiple steps:

- To begin mining a new block, a miner gathers transactions from the shared network and organizes them in a block.
- The miner will verify the blockchain’s prior hash value and deposit it with the transactions in the intended new block.
- The miner will obtain and save in the same block a variable called “nonce” (Figure 2). This variable value can be altered at any time by the miner.
- The miner will now investigate the network’s PoW puzzle. The problem consists of finding, for the whole new block, a special hash value starting with several zeros. This special hash value can be found by changing the nonce value which is the only parameter that the miner can modify. Once the miner discovers the same amount of beginning zeros for a given nonce value, he/she can broadcast the answer to the network and demonstrate that he/she succeeded in mining a new block. Note that the number of successive zeros indicates the mining difficulty level.

The nodes of type miners are also responsible for verifying all data contained within a block. To this end, the data of one block are saved with the shape of a Merkle tree, which represents a particular data structure in the form of a hash-based tree (Figure 2). Trees make data verification simple. Consider using the hash function of all transactions, not the structure of the Merkle tree. If a single transaction is altered, the entire hash result will be modified, making it impossible to detect the altered data. However, using the particular structure of the Merkle tree, we can see at any fraction of the tree which part delivers the erroneous hash value. Assume an attacker alters transaction Tx-3. As a result, we can easily detect that only the right side of the Merkle tree gives incorrect hash outputs. Because the hash values of Tx-3 and Tx-4 will be erroneous, we do not need to check Tx-1 and Tx-2. Consequently, the Merkle tree is extremely useful for data verification in peer-to-peer distributed systems [49].

## 4. Blockchain for Financial Activities

**Figure 3:**
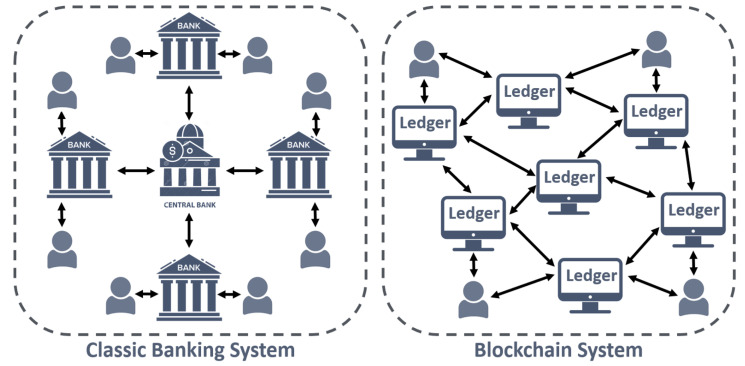
Illustration of the differences between the classic banking system (**left**) and the blockchain system (**right**).

Blockchain technology has been massively used in the financial and economic sectors [12,13]. For instance, it has been used for the settlement of financial market transactions, trade finance, insurance, real-time money transfer, cross-border payments, etc. Bitcoin was the world’s first decentralized cryptocurrency and a payment system not backed by a central bank. Without the need for an intermediary, transactions are performed directly between users through the P2P network [50] (Figure 3). Other cryptocurrencies, such as Bitcoin Cash, Ethereum, Ripple, and Dash are also available. The conventional cross-border payment system is based on the banking system, which has the disadvantages of being expensive, time-consuming, and less secure. However, by using blockchain to rebuild this payment system, all of these constraints may be efficiently solved [51]. Asset ownership (e.g., car, house, stocks, etc.) can be recorded, transferred, and verified using blockchain technology, as well as the integrity and validity of sensitive documents or data. The authors in [52] presented an extensive analysis of the differences between the main known cryptocurrencies in terms of release date, founder, the hash algorithm used, and the language used to develop it. Another interesting comparison between cryptocurrencies and the technology-based of blockchains and distributed ledgers behind them is found in [53].

Even though the use of blockchain technologies in the economic and financial fields appears to be highly promising, it still has a number of limitations [54,55]:

- Blockchain is too slow since it only allows for eight transactions per second. As a result, it has a significant disadvantage over the current third-party payment system Alipay [56], which can handle hundreds of transactions per second.
- If a private key or password is lost or disclosed, the blockchain system is impossible to recover, resulting in irreversible loss of consumer assets.
- Despite the fact that the blockchain is theoretically tough to crack violently, the risk of a data breach still exists.
- People still have a limited grasp and acceptance of blockchain technology, making it difficult to identify genuine and useful blockchain financial solutions.
- The lack of a centralized structure has made money laundering, fraud, and tax evasion more convenient, while also making supervision and control more complex.

## 5. Blockchain for Healthcare

**Figure 4:**
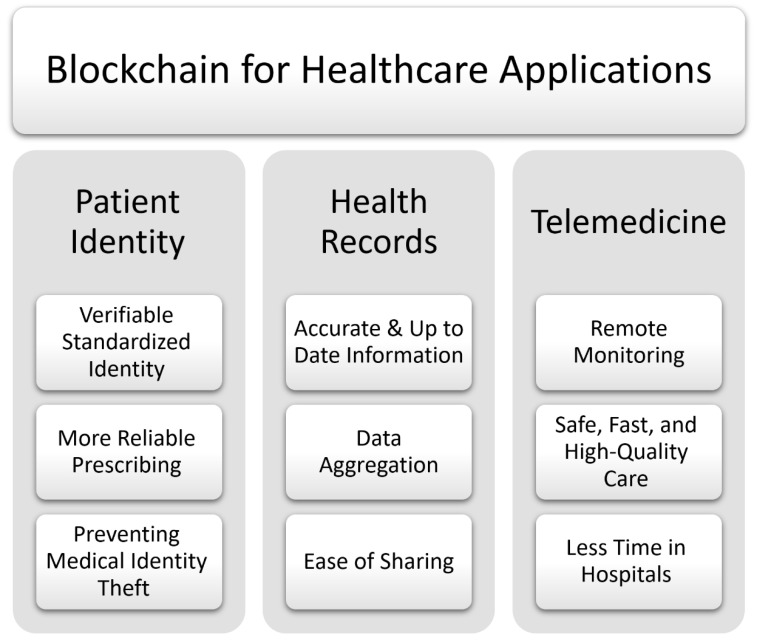
Blockchain for healthcare.

Despite the significance of medical data sharing, health systems usually compel a patient to collect and exchange his/her medical information with medical staff, either in print form or electronically on some storage devices. This method of distributing medical records is inefficient since it is slow, insecure, and incomplete. Moreover, it is “provider-centric” instead of being “patient-centric”. The inefficiency of this sharing method is mainly due to the lack of credibility between healthcare institutions and the lack of interoperability between the different IT platforms used by these institutions. According to [57], healthcare interoperability should cover three main levels, namely: foundational, structural, and semantic. This interoperability issue may be solved using blockchain technology [58,59]. Indeed, with blockchain implementation, patient medical information will be shared with necessary permissions using smart contracts for controlling operations, such as the change of viewership rights or the creation of new records. Next, we consider some examples of the use of blockchain technology in the healthcare field [60] (Figure 4):

- Patient identity: Patient identification [61] is a critical component of health information exchange. According to [61,62], medical errors cause 195,000 deaths every year in the USA, with identification problems accounting for 57% of the total number of errors. In such a situation, blockchain technology can impose a verifiable standardized identity for each patient through a universal patient index database, which may be shared between all healthcare facilities [63].
- Health records: Generally, the classical computerized centralized systems [64,65,66] do not address the root of the patient data sharing problem. However, thanks to blockchains [67,68,69], a patient may simply collect his/her medical history without asking for a copy from each provider he/she has visited. In this way, the blockchain technology allows for the creation of widely secure and accessible data distribution services that interface with different existing healthcare systems. Moreover, due to the use of a blockchain, data sharing between the patient and the doctor becomes easier and more secure [70].
- Telemedicine: Patients who are connected to the internet can avoid spending time in the healthcare center and receive fast treatment for small but critical problems. However, distant medical professionals may be unable to continuously access health data obtained during telemedicine treatment episodes, resulting in an incomplete medical history and putting the overall quality of care at risk. As a result, in this situation, the blockchain technology [71,72,73,74] can bridge the communication gap between different providers by eliminating the need for third-party authorities and empowering engaged participants to interact directly.

At this level, it is worth noting that the ability to store and handle large volumes of patient health data, ensure privacy and reduce operational costs are all requirements for implementing blockchain in healthcare [75,76,77].

## 6. Blockchain for Information Systems

**Figure 5:**
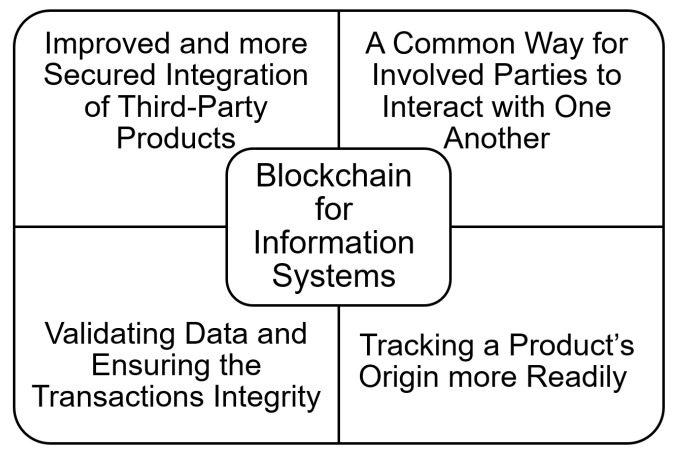
Blockchain for information systems.

An information system [78] is a collection of many different types of data that ensures the achievement of a business goal. Information systems are not really stand-alone IT business models. Integration with data and business processes, on the other hand, is a critical part of successful implementation. As a result, it is indeed easier to visualize the information system as a triangle. Processes, people, and computers are represented by the three elements of this triangle. To be successful, an information system must have all of these components working properly. The choice to integrate blockchain technology into information systems allows organizations to benefit from the vast array of applications and advantages that blockchain offers [79,80,81] (Figure 5).

Businesses, governments, and other organizations that maintain information systems sometimes rely on third-party agents or technologies to complete certain tasks. This necessitates the existence of a trust network among the partners involved, which is even more important when sensitive information is involved. Blockchain allows for improved and more secured integration of third-party products [82,83,84,85], while reducing the danger of revealing sensitive information to such parties. In addition, interoperability [86,87,88] fosters the promotion and acceptance of blockchain by providing a common way for involved agents to interact with one another via blockchain transaction ledgers and integrated networks, ensuring the validity of each engaged party.

Because blockchain is designed to be decentralized, it is an excellent contender for validating data and ensuring the transactions integrity. The adoption of the notion of “smart contract” [89,90,91,92] is one way to ensure transaction integrity. The purpose of smart contracts, as the name implies, is to allow the use of blockchains to ensure that two parties have an agreement being specifically composed into lines of code. This latter controls the execution, and exchanges are trackable and irreversible. If necessary, the blockchain could be utilized to resolve any disagreements that arise by confirming the authenticity of digital signatures in a safe, decentralized manner.

The fascinating utility of information systems, blockchain, and supply chain integration has been discovered for a range of businesses [93,94,95]. For example, many businesses consider product provenance to be critical. Blockchain can help track a product’s origins more readily due to local regulations, preferences, tax reductions, and other incentives to identify provenance tracking. The entire supply chain, including logistical factors, can benefit from provenance. An item can be officially confirmed at any time, and transactions cannot be falsified or altered for the purpose of deceiving the final consumers of the products [96,97,98].

In conclusion, there are several considerable advantages to the use of the blockchain technology in the commercial world. However, there is a real significant risk that for many small- and medium-sized enterprises, the overhead costs of implementing integrated blockchain technology would be prohibitive and almost infeasible.

## 7. Blockchain for Wireless Networks

Wireless applications, such as broadband internet connections, mobile smartphones, and internet of vehicles [99,100] all require radio spectrum [101], which is precious and restricted resources. Wireless networks, such as cellular and Wi-Fi, are the most cost-effective ways to provide broadband internet access, particularly in low-income areas and emerging nations. As a result, diverse spectrum management regimes are needed to optimize advantages from the utilization of the available spectrum by mandating efficient spectrum usage while minimizing interference between consumers [102]. The traditional spectrum management regime has two major drawbacks. First, large portions of the licensed spectrum are underused. Second, this command-and-control spectrum management regime is slow to respond to market and technology changes [103]. Spectrum sensing [104], supporting secondary spectrum trading marketplaces [105], spectrum sharing [106], and policy enforcement [107] are all possible uses for the blockchain technology in spectrum management [108].

Blockchain technology may be used to create a secure spectrum sensing system as well as enable collaborative sensing, both of which improve the accuracy of spectrum sensing data. Mobile network operators can use spectrum sensing to combine available empty frequencies with their licensed frequencies to boost network capacity. Collaborative sensing, which includes fusing the sensing findings from a number of secondary sensors or users, can ensure the efficiency of spectrum sensing outcomes. The blockchain was first used as a peer-to-peer payment system. As a result, it naturally lends itself to the creation of a full-spectrum payment system based on digital currency that can be quickly converted to fiat currency. The blockchain technology can be used to accomplish the many functions of a geolocation database as well as the needs of spectrum management. The use of blockchain to actively store information about unoccupied spectrum bands and user geolocations is expected to increase spectrum access and utilization efficiency as well.

A secure spectrum sensing technique based on blockchain is presented in [109] to increase the energy efficiency and sensing accuracy of cognitive wireless networks at the same time. The mechanism can adapt to changes in the environment and adjust the number of nodes engaging in cooperative sensing in real-time, as well as evaluate the dependability of sensing nodes in real-time and calculate the node’s trust value using an evaluation algorithm. Not only does the system record each node’s energy consumption and sensing performance, but it also remembers the trust value of a single node. The trust value is recorded in the blockchain’s reliability list, which is encrypted by the blockchain’s management center to ensure that each node matches its own trust value. The suggested algorithm in this research may take into account both energy efficiency and sensing accuracy, extending the working life of cognitive wireless networks, according to experimental data.

Blockchain technology and reputation system were introduced into the spectrum sensing method in this research. A new secure spectrum sensing approach is presented. The user’s direct reputation and referral reputation are both evaluated in this security sensing method. When a cooperative node asks for access to a certain frequency band, it must first determine whether the band is available. It will send a suggestion request to the fusion center if it is unresponsive. The sensing findings are more accurate in order to prevent collusion attacks and malicious node behaviors. The historical sensing records in the database and the distance of interaction history are regarded as a public ledger using blockchain technology, which can be shared by each neighbor node and no node in this situation can change the ledger information.

**Figure 6:**
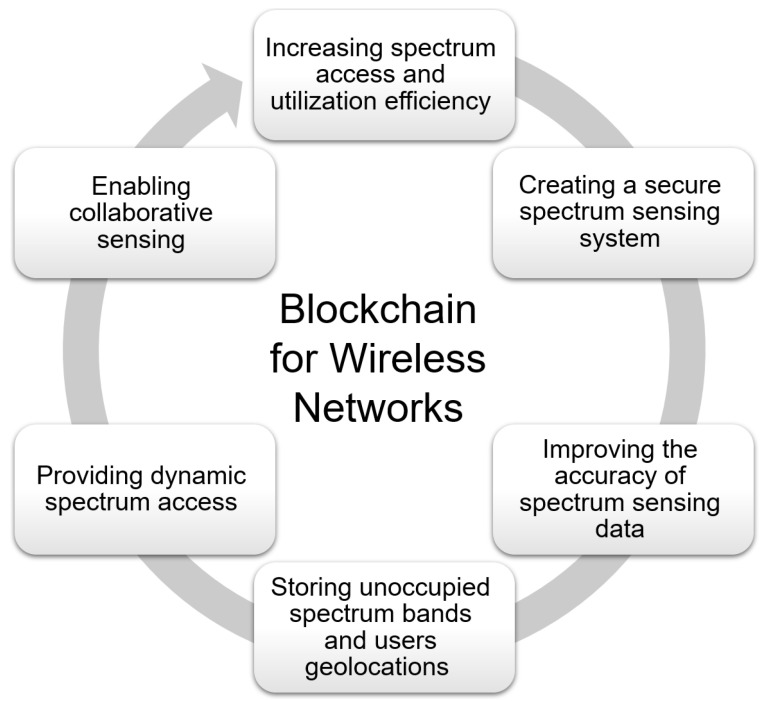
Blockchain for wireless networks.

Spectrum management using blockchains is a new application with a lot of opportunities and challenges. Spectrum sensing and geo-location databases are the two main technologies used for providing dynamic spectrum access. Previously, these approaches were viewed as separate strategies in previous research. Because blockchain is a database technology, it may be used to create a unified method in which spectrum sensing techniques and geolocation database technology work in tandem. A more robust dynamic spectrum management framework will arise from combining these two spectrum access strategies. It is also necessary to investigate the integration of blockchains with the communication networks. The blockchain network could be set up as an overlay on top of the communication network, allowing communication network nodes to operate as complete nodes on the blockchain network. This network structure, however, is energy-intensive and necessitates a specialized control channel for transferring blocks and transactions over blockchain networks [110]. The possible applications of blockchain technology for wireless networks are illustrated in Figure 6.

## 8. Blockchain for Internet of Things

The Internet of Things (IoT) [111,112,113] is the linking of smart devices for data collection and intelligent decision-making. Yet, IoT is prone to privacy and security risks due to the absence of inherent security measures. The dispersed and centralized architecture of the Internet of Things is a significant challenge [114,115,116]. Every node in an redIoT infrastructure is typically a potential point of weakness that could be used to start cyber assaults. Data confidentiality and authentication are other continuous and serious threats. IoT data could be hacked and misused if data security is not established [117]. Data integrity is another issue for IoT. Decision support systems are one of the most important IoT applications. As a result, protecting the system from injection attacks, which attempt to insert bogus measures and, thus, impact decision-making, is critical. For automated systems, such as manufacturing sectors and vehicular networks [118], which handle real-time data, availability is crucial. The inclusion of a publicly verifiable audit trail that is not reliant on a trusted third-party is essential, as it addresses all of these issues. Blockchain may assist in solving major security concerns in IoT with its “security by construction” feature [119,120].

Blockchain is the final piece of the puzzle in resolving IoT privacy and dependability issues. The blockchain’s inherent trustless, autonomous, and decentralized characteristics make it suited for use in a variety of scenarios. The blockchain technology, for example, may store a permanent record of smart gadgets [121,122]. Furthermore, the implementation of smart contracts may allow smart devices to perform autonomously, avoiding the need for human control or centralized authority. In addition, blockchain can establish a secure means for smart devices to communicate with one another [123,124].

The contribution in [125] can be viewed as a generic solution that can be used in any field of the IoT environment. Indeed, the authors of this paper developed a mechanism that would allow sensors to trade Bitcoin for data. Every node has a unique address that corresponds to the Bitcoin pub-key. When a user needs data from a sensor after locating it in a sensor repository, he sends a transaction directed to that sensor’s public key. The sensor will reply by sending a transaction containing data to the client. This strategy is an extension of the solution provided in [126]. The Enigma framework [127] offers yet another intriguing solution. The latter makes use of a completely comparable concept—distributing data over multiple nodes while separating data from its references. Furthermore, in addition to making it difficult to reconstruct the original form of data, Enigma offers an extra layer of protection by encrypting such data chunks. As a result, Enigma is a P2P network that allows several participants to store and process data at the same time while maintaining privacy.

**Figure 7:**
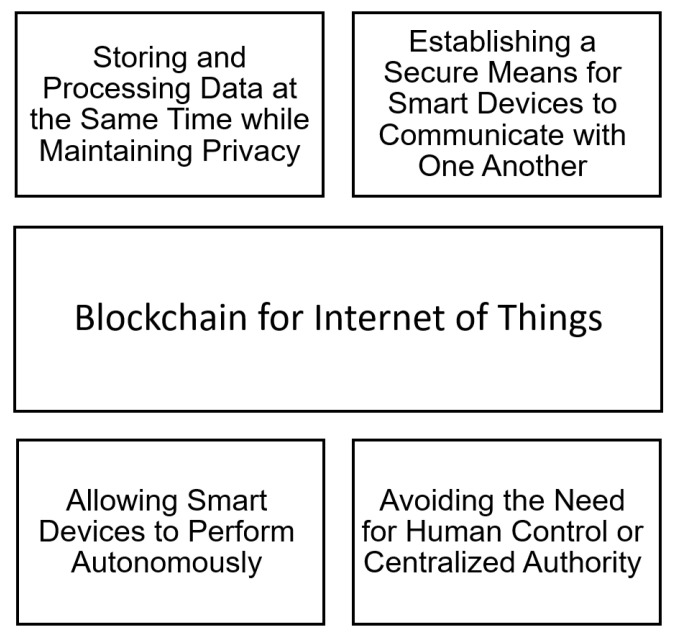
Blockchain for Internet of Things.

To summarize, the usage of blockchain for IoT applications provides excellent levels of security, which prevent unwanted data access (Figure 7). Yet, scalability [128] is still an open question since the blockchain can grow in size over time, making it difficult to acquire and save the ledger.

## 9. Blockchain for Smart Grids

**Figure 8:**
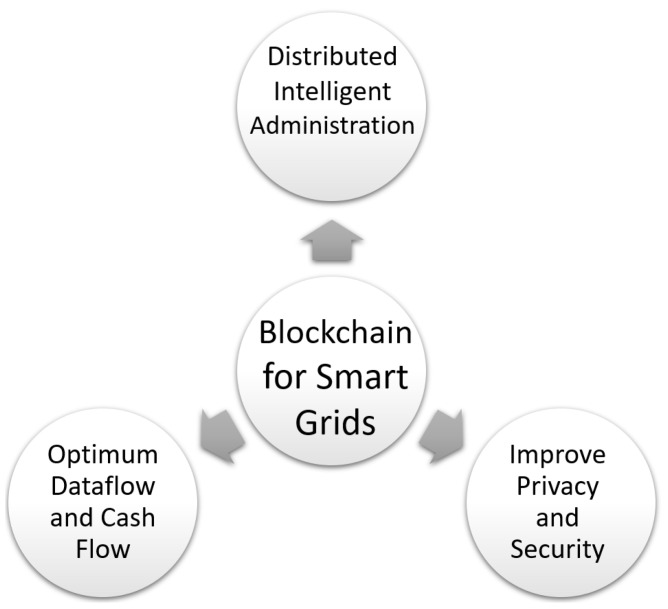
Blockchain for smart grids.

A smart grid [129,130,131,132] is a digital communications-based electrical network that provides for the two-way flow of electricity and data, and also the identification, reaction, and avoidance of changes in usage and other difficulties. Current smart grids integrate communication and control techniques into power networks, allowing for considerable gains in energy efficiency and system safety. Traditional centralized techniques of managing smart grids pose significant hurdles. For instance, the centralized control method creates a dangerous single point of failure for the whole grid. In addition, many security issues have been growing and external security assaults could result in significant financial losses. To overcome these limitations, the use of blockchain technologies is considered a good choice in several research and industrial projects [133,134,135]. Indeed the use of blockchain for smart grids may have the following advantages (Figure 8):

- The blockchain has the potential to turn centralized grid administration into distributed intelligent administration.
- In terms of energy trading, a smart grid with blockchain technology can achieve optimum data flow and cash flow.
- Because of its decentralization and fault tolerance, blockchain can dramatically improve the privacy and security of power grids.

Incorporating cryptocurrencies for payment is one of the most important applications of blockchain for smart grids. BASNederland was the first company to use Bitcoin as payment for energy bills. This prompted numerous additional companies to develop blockchain-based billing and metering services, with several of them offering incentives to consumers who pay with cryptocurrency. For instance: Bankymoon in South Africa using Bitcoin, Spectral, and Alliander in the Netherlands using Jouliette, PowerLedger in Australia using Sparkz, LO3Energy, and ConsenSys in the USA using Ethereum, etc.

Electric vehicles [136,137] can be thought of as mobile power grid terminals that perform key services. This is known as V2G technology, and it has the potential to increase the power grid’s reliability, efficiency, and stability. Electric vehicles, on the other hand, are not properly linked with smart grids, and there are a number of issues, such as energy shortages, security hazards, and data leakages. In this context, excessive charging loads and unsteady voltage in electric vehicles can be addressed with blockchain technology, as shown in [138,139]. In addition, using blockchain to connect smart grids and electric vehicles can lead to cost optimization through the use of smart contracts. Furthermore, using blockchain technology to connect smart grids and electric vehicles might reduce costs using smart contracts, as proposed in [140].

Although the use of blockchain technology for smart grids appears to be promising, as previously demonstrated, there are still hurdles in entirely converting to this new technology. For instance, re-architecting presents grid networks; implementing blockchain in the smart grid necessitates large infrastructural expenses, which will probably make grid operators hesitant to incorporate blockchains into their grid structures.

## 10. Blockchain for Governmental Services

Despite the fact that e-government initiatives have attempted to provide public services that are more straightforward, distributed, and adapted to the needs of inhabitants [141], they have never truly altered the functions of government agencies in record-keeping and management. One of the most important benefits of blockchain technology is the ability to promote direct interactions between government agencies, citizens, and businesses. As a result, blockchain technology has the potential to redefine how governments engage with individuals and each other, forcing public administrations to reconsider their roles in providing public services [142].

Governments might use this technology to take on supervisory functions over exchanges in a blockchain-based infrastructure. Blockchain has the potential to eliminate a considerable portion of the administrative functions that governments currently play in society, necessitating a shift in the governance of (public) service supply. This has the potential to change existing institutional frameworks, such as legal and public institutions [143].

**Figure 9:**
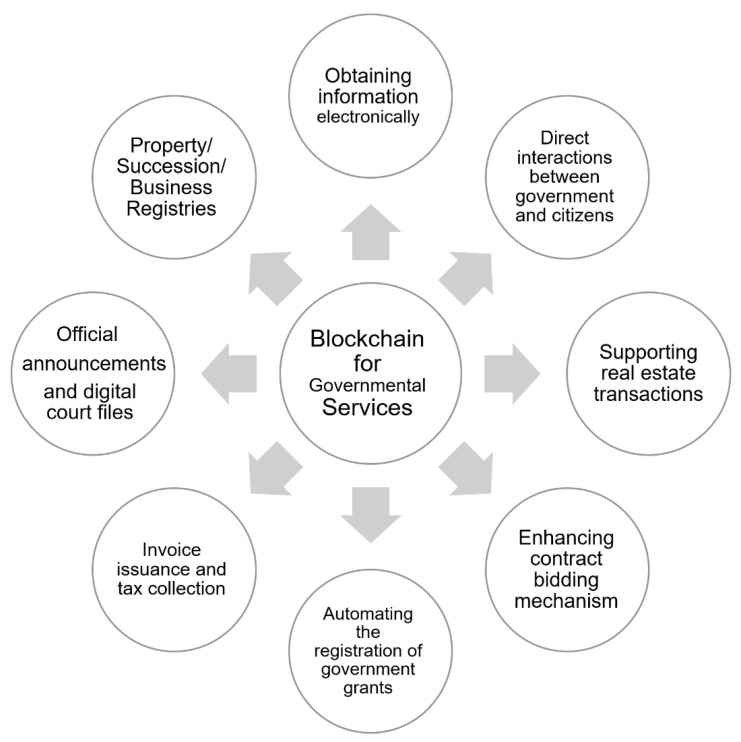
Blockchain for governmental services.

Next, we provide a short overview of the adoption of blockchain technology by different governments in the world (Figure 9):

- China: the Chinese government declared that it would begin employing blockchains in invoice issuance and tax collection.
- Japan: The Japanese government announced that it will be experimenting with a blockchain-based system for handling government tenders. The technology consists of allowing users to obtain information electronically, such as tax payment documents.
- USA: the US government was looking for contractors to evaluate how blockchain technology may be incorporated into its contract bidding mechanism.
- Britain: The incorporation of blockchain technology into governmental operations in the United Kingdom was offered as an interesting case study. The main concept behind blockchain use is to automate the registration and payment of government grants and perks.
- Estonia: blockchain technology has been integrated by the Estonian government in official announcements, digital court files, property registries, succession registries, business registries, etc.
- Sweden: the Swedish government has begun to explore the use of blockchain technology to support real estate transactions.

More research on the influences of these blockchain topologies on the technology–institution interface is required. Adopting blockchain technology for public services could result in not just a shift in the function of governments, but also a loss of jobs and a worsening of the digital divide. To minimize unforeseen repercussions when using this technology in the public sector, researchers should conduct research to compile a list of these effects. Finally, a study into public administration opinions toward blockchain technology could hasten its implementation.

## 11. Blockchain for Military and Defense

**Figure 10:**
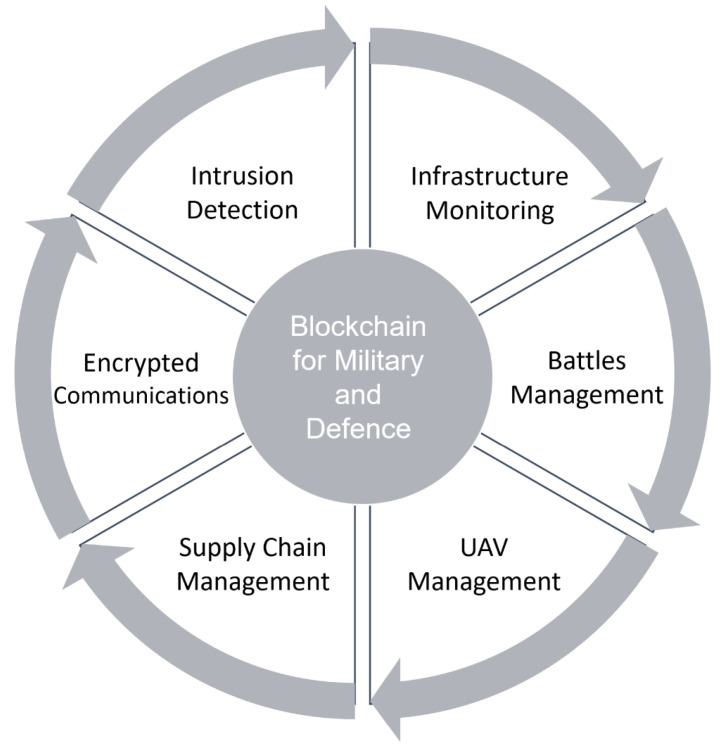
Blockchain for military and defense.

Military leaders who embraced cyber technology in the 1990s and early 2000s are now attempting to address the massive vulnerabilities that those same digital technologies produced [144,145]. Decades of hacking and exploiting cyber security systems have repeatedly proven how a determined cyber attacker may compromise military and civilian networks. The threat of sophisticated weapon systems being harmed or disabled by non-kinetic impacts have forced militaries to develop a long-term and ideally cost-effective defense for military systems [146]. Blockchain, and its as-yet-untested military uses, have the ability to shift the security vulnerabilities of some cyber systems from a single-point-of-failure vulnerability model, in which an attacker only needs to compromise one node to violate the system, to a majority-compromised vulnerability model, in which a malicious actor cannot exploit a single point of failure. The adoption of blockchain in the military field may cover the following aspects (Figure 10): (1) intrusion detection; (2) infrastructure monitoring; (3) battles management; (4) UAV management; (5) supply chain management; (6) encrypted communications.

The work presented in [147] proposes an interesting comparison of the adoption of blockchain technology by three of the strongest armed forces in the world:

- USA: outside of the realm of cryptocurrencies, US military conversations have centered on improving data resiliency, with the premise that the US military could eliminate data compromise and corruption as threats to its data, and that the blockchain technology might act as a cyber security shield.
- Russia: the Russian Ministry of Defense announced the creation of a research laboratory tasked with establishing a blockchain system for detecting and mitigating cyber attacks [148] on crucial military digital infrastructure.
- China: the interest in military applications of the blockchain technology in China concentrated on equipment management, professional learning, logistics, and the conversion of commercial information technologies into defense programs.

Even though the military applications built on top of the blockchain, so far, do not seem to be completely ready for use. Defense logistics and data security are likely to be the applications that will be concretely implemented for the military blockchain in the near future. On the other hand, the adoption of blockchain by the world’s strongest militaries is somewhat paradoxical. Indeed, while blockchain has the potential to share governance among citizens and guarantee more individual liberties, for the time being, the most centralized human organizations are committed to using this same technology to create a decentralized technology for military and defense applications.

## 12. Open Challenges

Many industrial unresolved problems need to be addressed and examined further in order to develop more usable and successful blockchain-based applications. In what follows, we discuss the main open problems.

- An in-depth study of the blockchain-based solution benefits: When applied to replace existing solutions [149], blockchain is a new technology that has the potential to destabilize the market, by introducing revolutionary ways that may transform society [150]. As a result, it is critical to establish whether a blockchain is truly required for a given application [151].
- Proper implementation: Blockchain is a general-purpose method of data manipulation that may be used in a variety of systems for various reasons, as long as its implementation has some degree of comprehension or maturity regarding its importance as well as the trade-offs. Indeed, the blockchain as a technology has various architectures, and different transaction processes; thus, its implementation is not a straightforward operation. Hence, its incorporation in different applications requires an in-depth and comprehensive study [152].
- Standard testing mechanism: another challenge faced when adopting a blockchain-based application is the need for a standard testing mechanism.
- Resilience to security risks: The resilience to security risks needs to be formally proved. With large-scale applications, the blockchain may face malfunctioning due to the system design or cyberattacks that intend to compromise its security.
- Scalability: This issue is raised basically from the fact that blockchain-based transactions are very slow to be processed and verified. Processing the transactions depend on the performance of the processing system. In [153], limitations of the proposed scaling methods are pointed out.
- Integration with other systems: This issue is a straightforward impact for organizations willing to adopt blockchain-enabled solutions. Indeed, the integration process will imply costs related to infrastructure change, trained staff, specialized developers, and management expectations [153].
- Energy challenges: the use of blockchain will undoubtedly require energy consumption much higher than the usual one. This challenge becomes an environmental issue when the energy used exceeds the load power and the equipment is fully utilized [154].
- Regulatory issues: regulations are of extreme importance to generalize and accept the use of blockchain-enabled solutions.
- Storage: The integration of blockchain with data-intensive applications, such as those based on the IoT, raises the problem of data storage. Indeed, blockchain stores data into blocks that cannot support large volumes of data. The authors in [7] proposed a hybrid architecture that combines blockchain with a decentralized database called IPFS. Another solution involves storing blocks in the cloud to benefit the extensible characteristic of the cloud, as proposed in [155].

In Table 3, we provide a summary of the main findings regarding the challenges associated with the use of the blockchain technology in the different considered fields. The abbreviation GDPR stands for “General Data Protection Regulation” and the abbreviation HIPAA for “Health Insurance Portability and Accountability Act”, respectively.

## 13. Conclusions

In this paper, we shed light on recent studies related to the incorporation of blockchain technology in modern applications, namely: financial activities; healthcare; information systems; wireless networks; Internet of Things (IoT); smart grids; governmental services; and military and defense. For each field, we provided related examples for the use of blockchain technology, while focusing on corresponding benefits, limitations, and challenges. The reviewed solutions are summarized in Table 4.

Blockchain is a revolutionary and exciting technology with enormous potential for usage in a wide range of modern applications. However, before the benefits of blockchain can be completely realized, a number of concerns and challenges must be addressed. One approach to addressing blockchain’s low throughput is to create new architectures and operational protocols for the system. The blockchain data, for example, may not be duplicated in every node in the network; instead, only the powerful nodes maintain a copy of the blockchain, while other light nodes simply save the block headers or do not save any data at all. To close the performance gap between a blockchain system and a typical database system, lightweight consensus techniques are also required.

While vertical and horizontal scaling of a blockchain system can help with scalability concerns, another research strategy is an interconnected multi-blockchain hierarchical structure with internal interconnections. Other approaches to reduce the amount of in-chain transactions could exist. Some transactions, for example, could be carried out directly between the parties without passing through the blockchain network; hence, enhancing blockchain scalability. Maintaining data security and privacy is difficult since all transactions committed to a blockchain are visible to all participants. Providing data auditability, on the other hand, may result in the loss of data and user anonymity. Manufacturing and enterprise solution data may have tremendous commercial value. As a result, in blockchain-based smart manufacturing systems, security and privacy are critical concerns. Before blockchain technology can be used on a broad basis, these and other security and privacy concerns must be addressed.

For more efficient, scalable, and secured blockchain industrial uses, additional work in the future is required. For instance, it will be interesting to investigate how machine learning (ML) techniques [175,176,177] may be used in the context of blockchain technology to increase security levels and the performances of blockchain-based systems. It will also be extremely useful to apply some formal testing techniques for blockchain-based solutions to improve their quality and increase their robustness [178,179,180].

## Figures and Tables

**Table 1:** Some examples of the use of blockchain technology in different fields.

Domain	Sub-Domains	Details
Finance [11,12,13]	Crowdfunding	Without the exorbitant fees charged by lawyers, creators obtain greater support for their initiatives with cheaper fees and overall costs.
	Money transfer	Companies attempt to address a variety of concerns with this technology, including high transfer costs, limited money distribution methods, etc.
Healthcare [14,15,16]	Patient-centric health records	Developing a blockchain-based medical record system that can serve as a single, encompassing representation of a patient’s data.
	Staff credential verification	Blockchain technology can be used to track the experiences of medical experts, allowing trustworthy medical institutions to document the credentials of their employees.
Information systems [17,18,19]	Preserving data integrity	The blockchain provides a secure, autonomous, and cost-effective proof-of-concept system that ensures that entries cannot be removed or changed.
	Cost efficiency and accuracy	Blockchain technology can reduce costs and increase accuracy while exchanging and storing vast amounts of data.
Wireless networks [20,21,22]	Security	Blockchain allows for secure communication with advanced wireless network technologies, such as edge computing, network slicing, open-source APIs, virtualization, etc.
	Access control	In wireless networks, blockchain technology provides a technique for anonymous access control.
Internet of Things [23,24,25]	Enhanced security	Blockchain offers a layer of security by encrypting data, eliminating single points of failure, and allowing users to rapidly discover the weakest link in a network.
	Reduced costs	The entire ecosystem may be made proactive at a lower cost by automating transaction validation and processing procedures on blockchain.
Smart Grids [26,27,28]	Renewable energy	To avoid double-counting, renewable energy certificates are recorded and awarded in real-time and automatically.
	Peer-to-peer trading	Automated smart contracts are used to sell excess renewable energy to other network participants.
Governmental services [29,30]	Registries	Using blockchain-based distributed ledgers to manage registries give the necessary transparencies to reduce fraud while also allowing for real-time modifications.
	Administration	Blockchain-based administration solutions allow for real-time collaboration across a wide range of stakeholders while also providing the necessary transparency.
Military and defense [31,32]	Marine aviation	Better tracking of aircraft replacement components, resulting in decreased operational costs.
	Logistics, procurement, and finance	The blockchain may be used to manage and register goods and services and it can be used to verify and register all financial transactions, improving efficiency.

**Table 2:** Summary of related surveys.

Ref.	Year	Domain	Goals	Limitations
[33]	2022	Blockchain for smart grid and energy trading	An assessment of blockchain applications in smart grids with regard to cyber security perceptions and energy data protections.	The authors concentrated only on security aspects and neglected other possible issues related to the use of blockchain technology.
[34]	2022	Blockchain for IoT systems	A survey and tutorial on blockchain applications, advancements, solutions, obstacles, and future research objectives for IoT systems.	The authors focused on a single application of blockchain technology (in the field of IoT systems).
[35]	2022	Blockchain for manufacturing supply chain and logistics	Comprehensive overview of blockchain technology’s role in tackling supply chain and logistics-related concerns.	The authors focused on a single application of blockchain technology (in the field of manufacturing supply chain and logistics).
[36]	2021	Approaches toward blockchain innovation	Overview of blockchain and its importance in the sectors of smart healthcare, smart grid, and smart financial systems.	Only a few applications of blockchain technology were considered and a few challenges were covered.
[37]	2021	Blockchain for transportation systems	A survey on the use of blockchain technology for improving the operation and security of transportation systems.	Only one application of blockchain technology was considered and few challenges were covered.
[38]	2021	Blockchain evolution	In-depth examination of blockchain technology’s evolution, architecture, development frameworks, and security concerns.	Adoption of a generic approach concerning the use of the blockchain; no specific application domains were covered.
[39]	2020	Blockchain-based smart systems	Comprehensive overview of blockchain technology’s applications and use cases for securing and trusting smart systems.	Few details provided concerning the application fields and the corresponding challenges.
[40]	2020	Blockchain for Industry 4.0	A comprehensive review on blockchain in Industry 4.0 architectures, techniques, applications, and challenges.	A limited number of issues and challenges covered, such as throughput and scalability, system integration, and privacy and security.
[41]	2020	Blockchain-based protection of multimedia	Taxonomy incorporating technical and application information for constructing blockchain-based multimedia copyright protection.	Not enough details about possible challenges and eventual issues related to this topic have been provided.

**Table 3:** Summary of the main challenges associated with the use of blockchain technology.

Domain	Scalability	Regulations	Security	Resources and Architecture	Interoperability
Financial activities [12,156]	The huge gap with the current third-party fast payment systems	Difficulty in supervising and managing, especially internationally	Vulnerabilities related to hacking and other cyberattacks	The slowness of cryptocurrency transaction processing and the high costs	The integration of various payment systems
Healthcare [157,158,159]	The size of the blockchain database is growing continuously over time with the flowed medical records	Compliance with GDPR and HIPAA standards esp for privacy-preserving issues [160]	Healthcare data sharing and medical data access controls, authentication, non-repudiation of records [161]	IoT healthcare devices are computationally-limited while blockchain is energy-greedy with high bandwidth consumption	The integration of blockchain with existing health information technology (HIT)
Information systems [19,162]	The structure and maintainability of blockchain-based IS with large system companies	Legal and regulatory issues in a decentralized information systems and standards to transform the business process	Security vulnerabilities, such as the border gateway protocol (BGP) routing hijack attack in smart contracts and privacy issues [163]	Difficulty in implementing a distributed computing system for small or start-up businesses	Compatibility issues between implementations of existing platforms and cloud or edge computing architectures with blockchain
Wireless networks [164]	Different and increasing wireless networks, such as 5G [165], 6G [166], and in envisioned UAV networks [167]	Trust degrees among stakeholders and regulation requirements for different use cases in wireless networks	Data collection, filtering, and data sampling require security assurance and privacy protection [20]	Memory and resource consumption in large-scale networks are enormous	The heterogeneity demands of hyperconnected existence of ‘everything’ wireless networks
Internet of Things (IoT) [34,168]	The network size and transaction volume make scalable solutions in IoT challenging	Considerable regulatory uncertainties exist in many countries concerning blockchain	Security risks due to smart-contract bugs to defect prevention	Increasing computing power and energy for IoT devices validate the transactions	Cross platforms with various architectural designs and implementations
Smart grids [28,169,170,171]	Properly scale-up the platform to accommodate the requirements of the smart grid system	The current grid legal system does not support the trading of energy from consumers to consumers.	(1) Cybersecurity threats to energy data generated by grid members and processes. (2) Cyber-physical attacks [172]	The need of transaction rates as high as a few thousand per second	The integration of heterogeneous distributed energy resources at different voltage levels
Governmental services [143,173]	Large and complex networks with data management (digital identity, administration, voting, etc.)	The regulations of E-governmental blockchain services require intensive governmental efforts	Integrity verification, high availability requirements. Ensuring authentication and authorization	Energy-inefficient mechanisms in the governmental services when using blockchain	Different governmental systems require compatibility across various platforms for governmental services
Military and defense [174]	Increasing the military network that includes hundreds of sensors to collect and transfer data	(1) Standards and regulations for the military field. (2) Compliance with standards related to preserving privacy	Military operation requires high security mechanisms for data and privacy assurance	Minimum execution time for a transaction to meet the military objectives and minimize delays	Immense heterogeneous data in the aerospace and defense industry when dealing

**Table 4:** Summary of the main findings concerning the use of blockchain in different fields.

Domain	Papers	Main Applications	Limitations
Financial activities	[12,13,50,51,54,55]	(1) Settlement of financial market transactions; (2) trade finance; (3) insurance; (4) real-time money transfer; (5) cross-border payment.	(1) Too slow; (2) risk of irreversible loss of consumer assets; (3) risk of a data breach; (4) limited grasp and acceptance; (5) supervision is more complex.
Healthcare	[58,59,60,61,63,64,65,66,67,68,69,70,71,72,73,74]	(1) Verifiable standardized identity; (2) more reliable prescribing; (3) preventing medical identity theft; (4) accurate and up to date information; (5) data aggregation; (6) ease of sharing; (7) remote monitoring; (8) safe, fast, and high-quality care; (9) less time in hospitals.	(1) Storing large records may be inefficient and extremely expensive; (2) data in a is difficult to query, restricting clinical, statistical, and research applications.
Information systems	[79,80,81,82,83,84,85,86,87,88,89,90,91,92,93,94,95,96,97,98]	(1) Improved and more secured integration of third-party products; (2) a common way for involved parties to interact with one another; (3) validating data and ensuring the transaction integrity; (4) tracking a product’s origin more readily.	Overhead costs of implementing integrated blockchain technology would be prohibitive and almost infeasible.
Wireless networks	[99,100,102,103,104,105,106,107,108,110]	(1) Increasing spectrum access and utilization efficiency; (2) creating a secure spectrum sensing system; (3) improving the accuracy of spectra sensing data; (4) storing unoccupied spectrum bands and user geolocations; (5) providing dynamic spectrum access; (6) enabling collaborative sensing.	(1) Energy-intensive; (2) necessitates a specialized control channel for transferring blocks and transactions over blockchain networks.
Internet of Things (IoT)	[117,118,119,120,121,122,123,124,125,126,127]	(1) Storing and processing data at the same time while maintaining privacy; (2) establishing a secure means for smart devices to communicate with one another; (3) allowing smart devices to perform autonomously; (4) avoiding the need for human control or centralized authority.	Scalability is still an open question since the blockchain can grow in size over time, making it difficult to acquire and save the ledger.
Smart grids	[133,134,135,136,137,138,139,140]	(1) Distributed intelligent administration; (2) improve privacy and security; (3) optimum dataflow and cash flow.	Large infrastructural expenses needed.
Governmental services	[142,143]	(1) Obtaining information electronically; (2) direct interactions between government and citizens; (3) supporting real estate transactions; (4) enhancing contract bidding mechanism; (5) automating the registration of government grants; (6) invoice issuance and tax collection; (7) official announcements and digital court files; (8) property/ succession/ business registries.	(1) Lack of legal and regulatory support; (2) issue of acceptability and the need of a new governance model.
Military and defense	[146,147]	(1) Infrastructure monitoring; (2) battles management; (3) UAV management; (4) supply chain management; (5) encrypted communications; (6) intrusion detection.	(1) Not completely ready for use; (2) somewhat paradoxical with the fact that military and defense applications need to be managed in a centralized fashion.

## Data Availability

Not applicable.
